# Rare Occurrence of Double Outlet Right Ventricle and Cardiomyopathy on Dynamic Contrast-Enhanced Cardiac MRI: A Case Report

**DOI:** 10.7759/cureus.38346

**Published:** 2023-04-30

**Authors:** Tushar Kalekar, Purnachandra Lamghare, Suhas M, Aparna Prabhu

**Affiliations:** 1 Radiodiagnosis, Dr. D. Y. Patil Medical College, Hospital & Research Centre, Pune, IND; 2 Radiodiagnosis, Dr. D. Y. Patil Vidyapeeth, Pune, IND

**Keywords:** dorv: double outlet right ventricle, conotruncal anomaly, radiology, cardiac magnetic resonance imaging, cardiomyopathy

## Abstract

We present a case of a middle-aged man with recent onset of vague chest pain. On dynamic contrast-enhanced cardiac magnetic resonance (CMR) imaging, he was found to have a double outlet right ventricle (DORV) and restrictive/infiltrative cardiomyopathy. These two conditions are not known associations and could be two entirely different entities.

## Introduction

Double outlet right ventricle (DORV) is a rare congenital anomaly accounting for approximately 2% of congenital cardiac defects [1,2]. In this condition, both the pulmonary artery and aorta arise from the right ventricle and is a type of conotruncal anomaly. It almost always has a concurrent ventricular septal defect [3]. It is known to be associated with a few other congenital cardiac anomalies like coarctation of the aorta, congenital pulmonary stenosis, and right-sided aortic arch. 

Cardiomyopathy is a disease of the myocardium with associated mechanical and/or electrical cardiac dysfunction [4]. They frequently have a genetic etiology and may be systemic or exclusive to the heart. Infiltrative cardiomyopathy can be caused by a wide spectrum of genetic and acquired conditions. The infiltration of abnormal substances results in ventricular wall thickening, heart failure, and conduction abnormalities. Though electrocardiography and echocardiography are helpful, cardiac magnetic resonance (CMR) and nuclear imaging are preferred for the diagnosis [5]. Restrictive cardiomyopathy is characterized by a marked decrease in ventricular compliance [6]. 

We present a case of DORV in a middle-aged man. He was also diagnosed to have restrictive/infiltrative cardiomyopathy which could be a completely different entity. These concomitant conditions are rare, and not reported in the medical literature. He also had cardiomegaly, right ventricular hypertrophy and enlargement, and right atrial enlargement. Other findings were a narrow right ventricular outlet and an aortopulmonary septal defect close to the origin of the aorta. Tissue diagnosis for confirmation of the type of infiltrative cardiomyopathy was not done for the patient.

## Case presentation

A 44-year-old male presented with a history of vague chest pain and orthopnea for two weeks. The patient was hemodynamically stable. Troponin-I levels were normal (<10 pg/mL). The ECG showed P pulmonale, right ventricular strain pattern, and right bundle branch block (RBBB). The patient underwent 2D echocardiography which revealed dilated hypertrophied right ventricle with severe right ventricular outflow tract (RVOT) obstruction and a suspicious double-chambered right ventricle. The pulmonary valve was thickened. A D-shaped left ventricle was seen, likely due to right ventricular pressure overload. Good left ventricular systolic function was seen with a left ventricular ejection fraction of 60%. 

Then the patient underwent CMR imaging with dynamic contrast for further evaluation of the condition. It revealed mild cardiomegaly with dilatation and hypertrophy of the right ventricle and right atrium with moderate tricuspid regurgitation. The left ventricle was small. Thick irregular walls of the right ventricles and septae were seen. The narrowing was seen at the right ventricular outlet and the origin of the pulmonary artery (valvular), which had a common origin of the aorta from the right and left ventricle, suggestive of a double-chambered right ventricle. The pulmonary artery measured 19 mm and was normal in caliber. Conotruncal abnormality was seen in the form of a small proximal aortopulmonary septal defect (aortopulmonary window) 11 mm from aortic origin. Mild hypokinesia of both ventricles was seen. The heterogeneous hyperintense signal was seen in the walls of the right ventricular and interventricular septum myocardium. On the post-contrast scan, delayed scan heterogeneous and patchy enhancement was observed in almost the entire right ventricle, interventricular septum, and inferior wall of the left ventricle, suggestive of secondary infiltrative/restrictive cardiomyopathy. Mild hypokinesia of both ventricles was seen. There was no evidence of a filling defect/thrombus seen in the cardiac chambers. There was no evidence of infarction or scar in delayed post-contrast imaging. Left ventricular analysis showed end-diastolic volume (EDV) of 51.97 mL, end-systolic volume (ESV) of 15.23 mL, and systolic volume (SV) of 36.74 mL. Normal systolic function and a left ventricular ejection fraction (EF) of 70.7 % were seen. Aorta diameter measured 3.0 cm at the root, 2.8 cm at the arch, and 1.8 cm at descending aorta. The patient was on metoprolol succinate 25 mg once daily.

**Figure 1:**
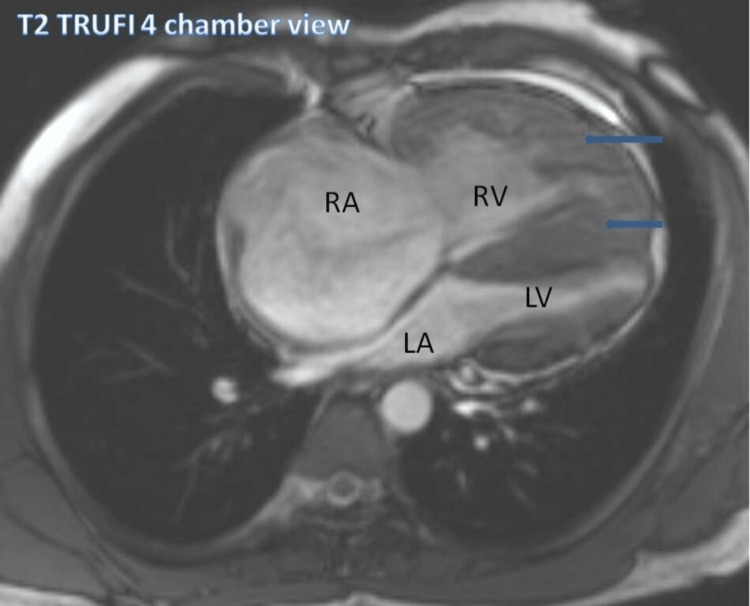
The T2 TRUFI four-chamber view Blue arrows point to the wall thickening of the right ventricle and interventricular septum. TRUFI: True fast imaging with steady-state free precession, RA: Right atrium, RV: Right ventricle, LA: Left atrium, LV: Left ventricle

Figure 1 shows the enlarged right ventricle and right atrium on the T2 true fast imaging with steady-state free precession (TRUFI) four-chamber view. There is marked thickening in the walls of the right ventricle and septum. The left ventricle is small.

**Figure 2:**
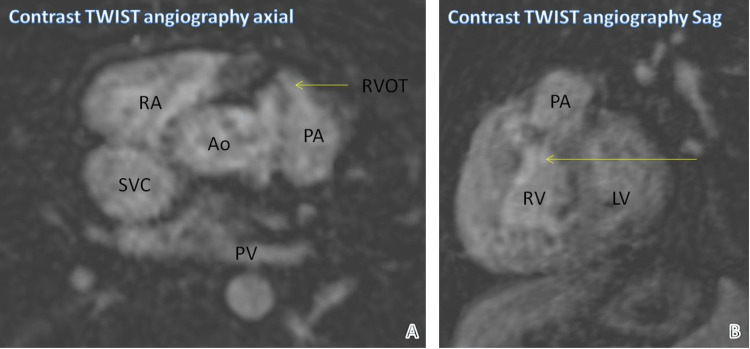
Narrow RVOT and origin of pulmonary artery Yellow arrows point to the narrow RVOT and the origin of the pulmonary artery. RVOT: Right ventricular outflow tract, Ao: Aorta, PA: Pulmonary artery, PV: Pulmonary vein, LV: Left ventricle, RA: Right atrium, RV: Right ventricle, SVC: Superior vena cava, TWIST: Time-resolved angiography with interleaved stochastic trajectories, Sag: Sagittal

Figure 2 shows reformatted axial and sagittal images of contrast time-resolved angiography with interleaved stochastic trajectories (TWIST) angiography. They demonstrate the narrow RVOT and the origin of the pulmonary artery.

**Figure 3:**
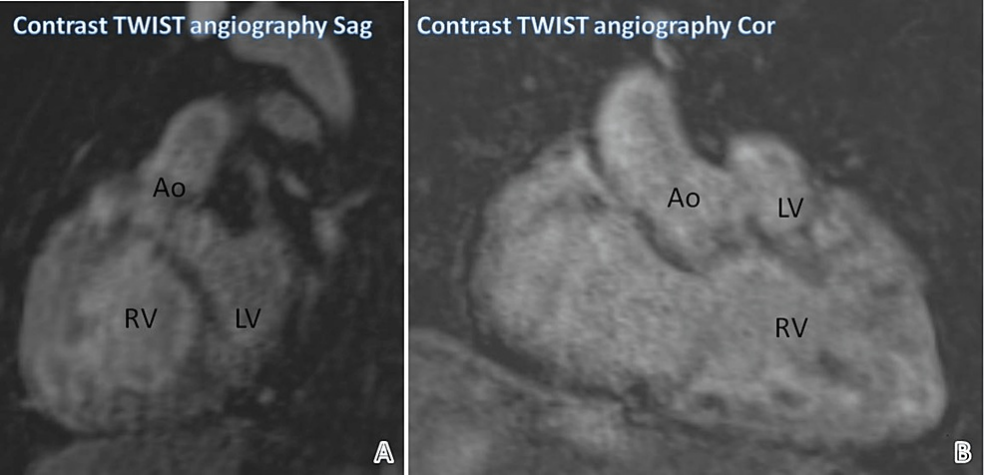
Origin of the aorta from both the right and left ventricle Ao: Aorta, LV: Left ventricle, RV: Right ventricle, TWIST: Time-resolved angiography with interleaved stochastic trajectories, Sag: Sagittal, Cor: Coronal

Figure 3 shows reformatted sagittal and coronal images of contrast TWIST angiography. They demonstrate the origin of the aorta from both the right and left ventricles.

**Figure 4:**
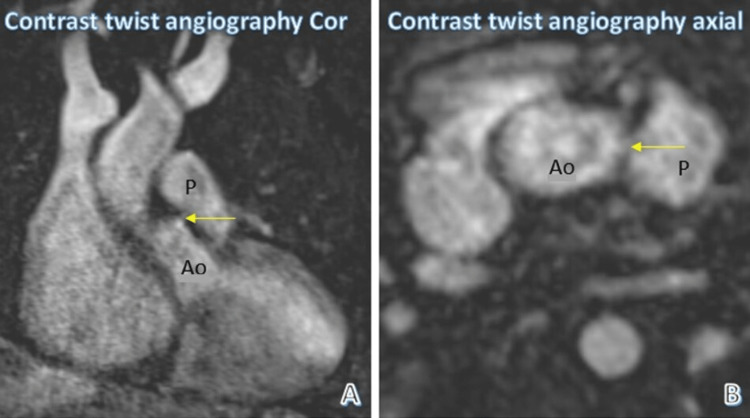
Aortopulmonary septal defect Yellow arrow points to the aortopulmonary septal defect. Ao: Aorta, P: Pulmonary artery, TWIST: Time-resolved angiography with interleaved stochastic trajectories, Cor: Coronal

Figure 4 shows reformatted coronal and axial images of contrast TWIST angiography. They demonstrate the small 2.5 mm aortopulmonary septal defect close to the origin of the aorta.

**Figure 5:**
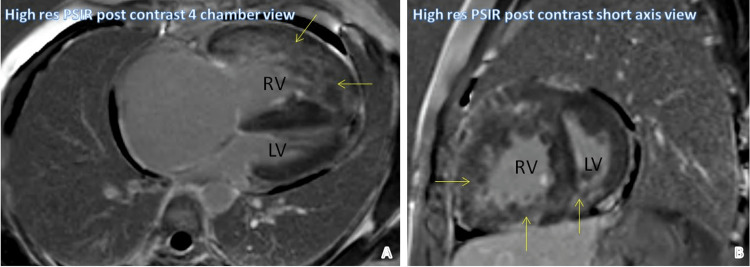
Cardiomyopathy Yellow arrows point to the patchy heterogeneous post-contrast enhancement of the myocardium involving almost the entire RV, part of the interventricular septum, and LV. RV: Right ventricle, LV: Left ventricle, PSIR: Phase-sensitive inversion recovery

Figure 5 shows high-resolution phase-sensitive inversion recovery (PSIR) post-contrast images in four-chamber and short-axis views. They demonstrate the patchy heterogeneous post-contrast enhancement of the myocardium involving almost the entire right ventricle, part of the interventricular septum, and left ventricle.

## Discussion

Double outlet right ventricle is a rare congenital anomaly where both aorta and pulmonary trunk arise from the right ventricle. It is classified as a conotruncal anomaly and accounts for approximately 2% of congenital cardiac defects [1,2]. Conotruncal anomalies are congenital defects resulting from abnormal formation and separation of the great vessels and outflow tracts of the heart [7]. It is known to have associations with chromosomal anomalies like trisomy 13, and trisomy 18; cardiovascular conditions like congenital cardiac anomalies like coarctation of the aorta, congenital pulmonary stenosis, right-sided aortic arch, anomalous pulmonary venous return, and tracheoesophageal fistula. Double outlet right ventricle is almost always associated with VSD and is divided into four types based on the location of the VSD and great vessels. Cardiovascular magnetic resonance imaging is an important modality for detecting these conditions. It allows for accurate delineation of cardiovascular anatomy and ventricular function assessment. This is useful for treatment planning and it can be supplemented with echocardiography findings [7]. 

Infiltrative cardiomyopathies occur as a result of the infiltration of abnormal substances, which can be due to genetic or acquired conditions. They occur in a wide variety of age groups and involvement of other systems is common given the systemic nature of the disease and gives vital clues to the diagnosis. Common types of infiltrative cardiomyopathies are cardiac amyloidosis, cardiac sarcoidosis, and hemochromatosis/iron overload cardiomyopathy. Other rarer genetic causes are Fabry disease, Danon disease, and Friedrich’s ataxia. The extent of the abnormality depends on the degree of infiltration and causes ventricular wall thickening, chamber dilatation, and conduction abnormalities. These changes result in the development of heart failure, ventricular arrhythmia, and atrioventricular (AV) block. Diastolic dysfunction with restrictive physiology predominates in the early stage, while adverse remodeling leads to systolic dysfunction in the advanced stages. Electrocardiography and echocardiography aid diagnosis, however, nuclear imaging and CMR imaging are preferred. Tissue diagnosis is done by endomyocardial biopsy or non-cardiac biopsy as in cardiac sarcoidosis. Treatment is based on etiology and extent of involvement and includes medications, cardiac devices, and transplantation. Most conditions have poor prognoses, with few exceptions like glycogen storage diseases where early diagnosis and treatment can be curative [5]. 

In amyloid cardiomyopathy, the classic finding on CMR is global transmural and subendocardial late gadolinium enhancement (LGE). The echocardiogram shows left ventricular and right ventricular hypertrophy. Endomyocardial biopsy is the gold standard for diagnosis. Cardiac sarcoidosis on CMR imaging shows patchy LGE predominantly in the left ventricular free wall and basal septum. Corticosteroids play an important role in treatment. Iron overload cardiomyopathy shows shortened T2 time on CMR imaging and are treated with phlebotomy/chelation [5]. 

Our patient showed DORV with narrow right ventricular outflow and aortopulmonary septal defect. He had developed cardiomegaly, right ventricular hypertrophy and enlargement, and right atrial enlargement. He showed patchy heterogeneous LGE of myocardium involving almost the entire right ventricle, part of the interventricular septum, and left ventricle which is likely to be due to infiltrative cardiomyopathy.

## Conclusions

Double outlet right ventricle is a rare congenital cardiac anomaly for which CMR imaging is an important modality for delineating the cardiovascular anatomy and assessing ventricular function. The DORV is known to have multiple associations with chromosomal anomalies and congenital cardiac anomalies. Infiltrative cardiomyopathies are a diverse group of cardiac diseases due to the deposition of abnormal substances in the heart, and their diagnosis and workup involve echocardiography, electrocardiography, CMR imaging, nuclear studies, and tissue diagnosis. Double outlet right ventricle and restrictive or infiltrative cardiomyopathy have no known associations, and there is a rare co-occurrence of these two different entities in our case.
